# Observation of non-Markovian micromechanical Brownian motion

**DOI:** 10.1038/ncomms8606

**Published:** 2015-07-28

**Authors:** S. Gröblacher, A. Trubarov, N. Prigge, G. D. Cole, M. Aspelmeyer, J. Eisert

**Affiliations:** 1 Kavli Institute of Nanoscience, Delft University of Technology, Delft 2628 CJ, The Netherlands; 2 Vienna Center for Quantum Science and Technology (VCQ), Faculty of Physics, University of Vienna, Vienna A-1090, Austria; 3 Dahlem Center for Complex Quantum Systems, Freie Universität Berlin, Berlin 14195, Germany

## Abstract

All physical systems are to some extent open and interacting with their environment. This insight, basic as it may seem, gives rise to the necessity of protecting quantum systems from decoherence in quantum technologies and is at the heart of the emergence of classical properties in quantum physics. The precise decoherence mechanisms, however, are often unknown for a given system. In this work, we make use of an opto-mechanical resonator to obtain key information about spectral densities of its condensed-matter heat bath. In sharp contrast to what is commonly assumed in high-temperature quantum Brownian motion describing the dynamics of the mechanical degree of freedom, based on a statistical analysis of the emitted light, it is shown that this spectral density is highly non-Ohmic, reflected by non-Markovian dynamics, which we quantify. We conclude by elaborating on further applications of opto-mechanical systems in open system identification.

## Abstract (web summary)

All quantum systems are connected to their environment, and this reduces their quantumness through decoherence. Here, the authors show that the interaction between a macroscale quantum system—a micromechanical oscillator—and its environment leads to non-Markovian Brownian motion

## Graphical abstract

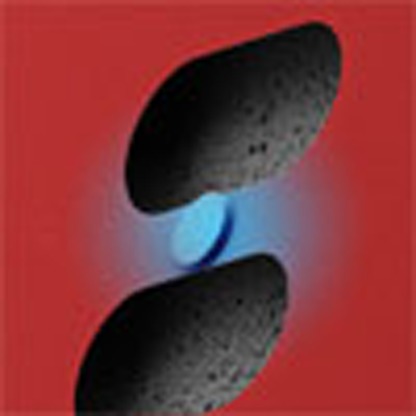

At the heart of understanding the emergence of a classical world from quantum theory is the insight that all macroscopic quantum systems are to some extent coupled to an environment and hence are open systems^1^,^2^,^3^,^4^. The associated loss of quantum coherence, that is, decoherence, is also detrimental for quantum information-processing applications. In contrast, properly engineered quantum noise can counteract decoherence and can even be used in robust quantum state generation^5^,^6^,^7^. To exploit the detailed dynamics of a quantum system, it is therefore crucial to obtain both good knowledge and control over its environment^8^,^9^,^10^. An explicit modelling of the environment, however, may often not be possible. In this case, simplifying assumptions concerning the nature of the underlying quantum noise are being made that do not necessarily hold for real devices. Micro- and nanomechanical resonators constitute prominent examples. They are now emerging as promising devices for quantum science^11^,^12^,^13^,^14^,^15^,^16^,^17^. Because of their complex solid-state nature, the properties of their intrinsic decoherence mechanisms have been the subject of intense research for decades^18^,^19^.

In this work, we present a method to reconstruct the relevant properties of the environment, that is, its spectral density, of the centre of mass motion of a micromechanical oscillator. We observe a clear signature of non-Markovian Brownian motion, which is in contrast to the current paradigm to treat the thermal environment of mechanical quantum resonators as fully Markovian. The presented technique, inspired by methods of system identification, can easily be transferred to other harmonic systems that are embedded in a complex environment, for example, electronic or nuclear spin states in a solid-state matrix^20^,^21^. Our results also open up a route for mechanical quantum state engineering via coupling to unorthodox reservoirs.

## Results

### Open quantum systems

To understand the role of the environment on a (quantum) mechanical system, let us first consider an isolated harmonic oscillator of bare frequency Ω and mass *m*. In the absence of any coupling, its centre of mass coordinate *q* will undergo undamped harmonic motion. In any real physical situation, however, the macroscopic degree of freedom of interest—here the centre of mass—will be coupled to some extent to a thermal bath of some temperature. Irrespective of the underlying microscopic mechanism, for example, phonon scattering in mechanical systems^22^ or electronic interactions in superconductors^23^, one can usually very well approximate the interaction with the thermal environment as a linear coupling to a bath of harmonic bosonic modes^24^. This is particularly true for high temperatures where finite bath degrees of freedom no longer significantly contribute. Such an interaction is described by


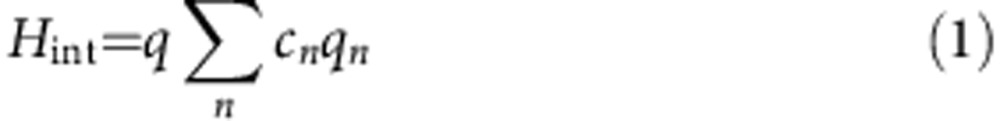


where *q*_*n*_ and *c*_*n*_ are the position and coupling strength of the *n*^th^ bath mode of mass *m*_*n*_ and frequency *ω*_*n*_, respectively. The dynamics of the system is fully determined by the spectral density of the thermal bath,


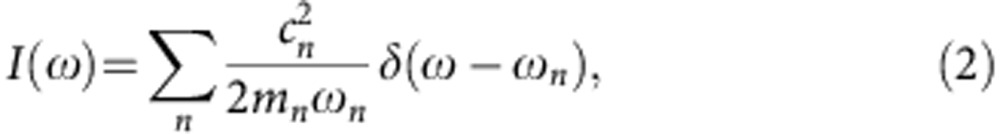


which governs how strongly the oscillator is coupled to specific modes of the environment. This spectral density directly determines the temporal correlations of the thermal driving force. As a consequence, the centre of mass experiences a quite drastic change in its motion: it becomes damped, in general in a rather intricate manner, and is shifted in its frequency. This quantum Brownian motion^25^,^26^ is one of the most paradigmatic models of decoherence in quantum theory^1^,^2^,^27^,^28^. It is this generic model for an unknown arbitrary spectral density that is the basis for our analysis.

All current theoretical studies on micro- and nanomechanical quantum systems make the explicit or implicit assumption that the decohering quantum dynamics is Markovian: this means that the open system dynamics is forgetful^29^,^30^,^31^,^32^. In this case the two-point correlation function of the thermal force equals *k*_*B*_*Tδ*(*t*−*t*′) and is hence uncorrelated in time. For a weakly damped mode at high temperatures (and in contrast to the situation in spin-Bose models^33^), such Markovian quantum dynamics is found for an Ohmic spectral density


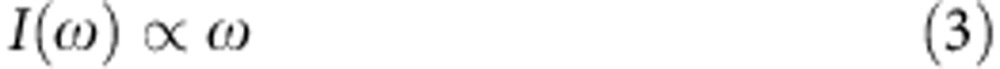


over large frequency ranges. For such damped harmonic systems in the high temperature limit, spectral densities other than Ohmic ones lead to deviations from Markovian evolutions. This is a widely known expectation^13^,^26^,^27^. In this work we precisely link properties of spectral densities with a quantitative measure of non-Markovianity.

In many solid-state systems, the Markov approximation has been found to be both theoretically plausible and experimentally valid to extraordinarily high precision^3^. Various loss mechanisms in mechanical resonators, however, are known to exhibit a strong frequency dependence^18^,^22^, which challenges the general validity of this approximation even for simple mechanical quantum devices. We introduce a straightforward test to directly characterize the spectral properties of the environment in the vicinity of the mechanical mode. Because of the complex solid-state architecture of these resonators, computing the spectral density from first principles seems a tedious, if not impossible, task with the exception of well-isolated loss mechanisms. Instead of making *a priori* assumptions about the dynamics, our approach is rather in the spirit of open system identification: we measure the properties that give rise to a quantitative estimate on the Markovian nature of the dynamics.

### Experimental set-up

Our approach relies on monitoring the mechanical motion with high sensitivity. We achieve this by weakly coupling the mechanics to an optical cavity field whose phase response encodes the mechanical motion^34^. We then make use of the fact that the shape of the bath spectral density affects the amplitude response of the mechanical resonator on thermal driving. Specifically, the experimentally accessible spectrum of the cavity output light for high temperatures is given by


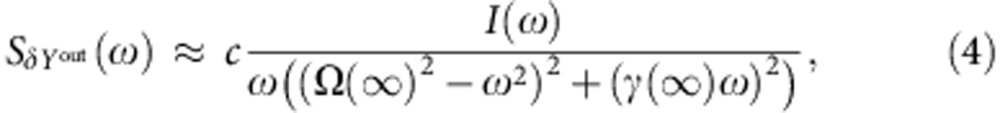


for a suitable constant *c*>0 (for details, see Supplementary Note 2). Here *δY*^out^ is the optical phase quadrature, which can be made a direct measure of the mechanical position quadrature *q* and which is obtained by optical homodyne readout, Ω(∞) is the renormalized mechanical frequency and *γ*(∞) is the effective asymptotic mechanical damping constant. The opto-mechanical device can hence be seen as an ultrasensitive black box measuring the spectral density.

**Figure 1:**
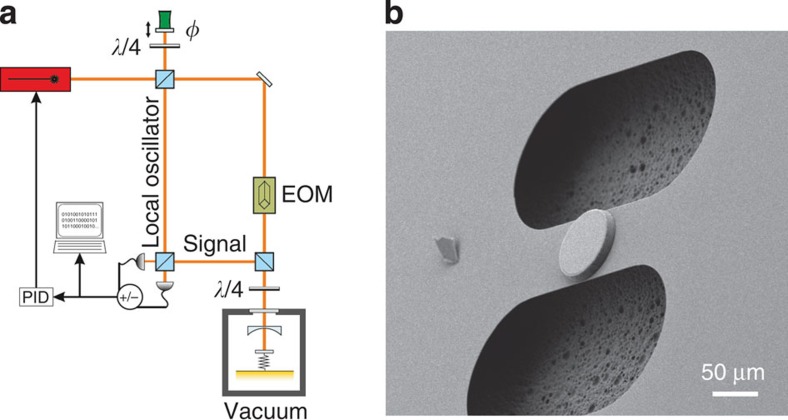
Sketch of the experiment. (**a**) The experimental set-up consists of a 1,064-nm Nd:YAG laser, which is split into a signal beam and a local oscillator (LO). The signal is phase-modulated with an electro-optical modulator (EOM) for Pound–Drever–Hall locking of the opto-mechanical cavity. In order to readout the phase of the signal beam acquired from the motion of the mechanical resonator, it is beaten with a strong LO on a beamsplitter and detected on two photodiodes. The phase *φ* between the LO and the signal is stabilized with the help of a mirror mounted on a piezo-ceramic actuator in order to only detect the phase quadrature of the signal field. The opto-mechanical cavity is kept at a pressure of <10^−3 ^mbar to avoid residual-gas damping of the mechanical motion. (**b**) Scanning electron microscope picture of the tested device.

**Figure 2:**
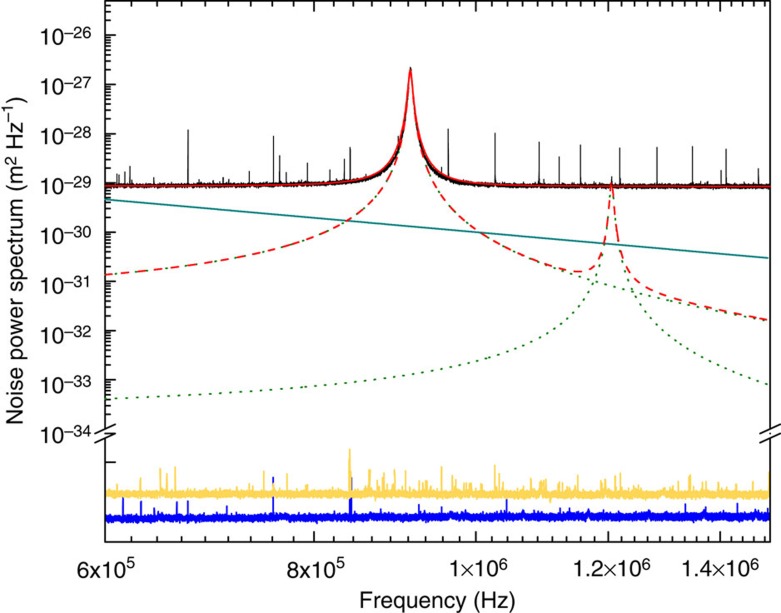
Noise power spectra. Depicted are the spectra obtained with the mechanics being part of the set-up (black; with a fit in red), with no mechanics (yellow), with no cavity (blue), a spectrum reflecting a sub-Ohmic spectral density *I*(*ω*)∝*ω*^−2^ (turquoise), the simulated sum (red dashed) and the simulated modes (green dotted). In our simulation we have assumed the mechanical Qs of the higher-order modes to be similar to the fundamental mode, which is in good agreement with typical experimental values. Note that for clarity the measurements of the additional noise (yellow and blue) are not to scale.

We demonstrate our analysis on a micromechanical resonator as shown in Fig. 1b. The device consists of a 1-μm-thick layer of Si_3_N_4_ and is 150 μm long and 50 μm wide. The 50-μm diameter, high-reflectivity (*R*>99.991%) mirror pad in its centre allows to use this resonator as a mechanically moving end mirror in a Fabry–Pérot cavity, as has been fabricated to explore the regime of cavity opto-mechanical coupling^35^,^36^ (for details on the fabrication process see ref. 37). In our case, the cavity finesse is intentionally kept low at *F*=2,300 by choosing a high-transmittivity input mirror for this experiment. This results in an amplitude cavity decay rate of *κ*=1.3 MHz (cavity length: 25 mm). By using a signal beam of 100 μW, we realize a sufficiently weak opto-mechanical coupling *g*≈40 kHz<<*κ*, such that the cavity field phase quadrature adiabatically follows the mechanical motion and hence *δY*^out^ is a reliable measure of *q*. The fundamental mechanical resonance frequency is *Ω*=2*π* × 914 kHz, with a mechanical quality *Q*-factor of ∼215 at room temperature. Optical homodyne detection of the outgoing cavity field finally yields the temporal phase quadrature fluctuations *δY*^out^(*t*), which are digitized to calculate the noise power spectrum *S*_*δY*out_(*ω*) (see Fig. 1a). All experiments have been performed in vacuum (background pressure <10^−3 ^mbar) to prevent the influence of fluidic damping. At the mentioned parameters for our experiment, we achieve a displacement sensitivity of ∼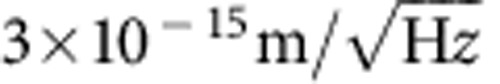 as is shown in Fig. 2. To exclude the possible influence of spurious background noise we have also characterized the noise power spectrum of the cavity field without a mechanical resonator. In our configuration this is possible because of the specific design of the chip comprising the micromechanical device, which holds several non-suspended mirror pads that can be accessed by translating the chip. The resulting noise power spectrum is flat and hence cavity noise cannot contribute to any non-Brownian spectral signal (see Fig. 2). Another possible spectral dependence could arise from the presence of higher-order mechanical modes, which are not taken into account in equation 4. A finite element analysis of our mechanical system reveals the next mechanical mode at *Ω*^(1)^=2*π* × 1.2 MHz. As can be seen from Fig. 2, the spectral overlap in the vicinity of *Ω* is many orders of magnitude below the measured signal and hence negligible.

### Spectral densities and non-Markovian dynamics

After characterizing the resonator, the final task to perform bath spectroscopy now reduces to assessing the statistical significance of a single assumption: namely that the spectral density is locally, that is, in the vicinity of an estimate of *Ω*, well described by


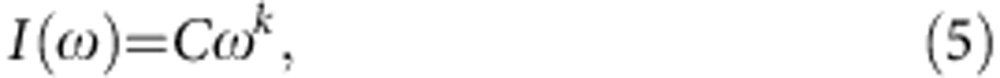


for some *C*>0 and *k*∈R, for *ω*∈[*ω*_min_,*ω*_max_]. A value of *k*=1 corresponds to an Ohmic environment, *k*>1 to a supra-Ohmic, and *k*<1 to a sub-Ohmic environment. This is the common classification of spectral densities^26^. For a slowly varying spectral density, however, what largely determines the long-time dynamics is the slope of the spectral density in the vicinity of *Ω*. Indeed, for this analysis to be valid, we do not have to make a global model for the spectral density—information that is experimentally inaccessible anyway—but merely for the local frequency dependence. We accompany this analysis with an analytical assessment in notes 2 and 3 of the Supplementary Material.

The starting point of this analysis is equation 4. From the homodyne measurement, samples of statistically independent subsets of time series are formed, and data sets are obtained as Fourier transforms thereof. For each of these independently distributed Fourier transforms, one identifies the optimal *k* in equation 4 with *I*(*ω*)=*Cω*^*k*^ that minimizes the least square deviation within a suitable frequency interval [*ω*_min_, *ω*_max_] centred around *Ω*. Here *ω*_min_=885 kHz and *ω*_max_=945 kHz are chosen; however, the results are largely independent of that choice. Interestingly, it is the comparably low mechanical *Q*-factor that allows for the assessment of a relatively large frequency interval. For each individual data set, several different values of the power *k* are compatible with the data, which is an unsurprising finding in the light of the presence of noise in the data. Given the large data set that is available, however, one can arrive at an estimate of the optimal coefficient *k* with large statistical significance.

**Figure 3:**
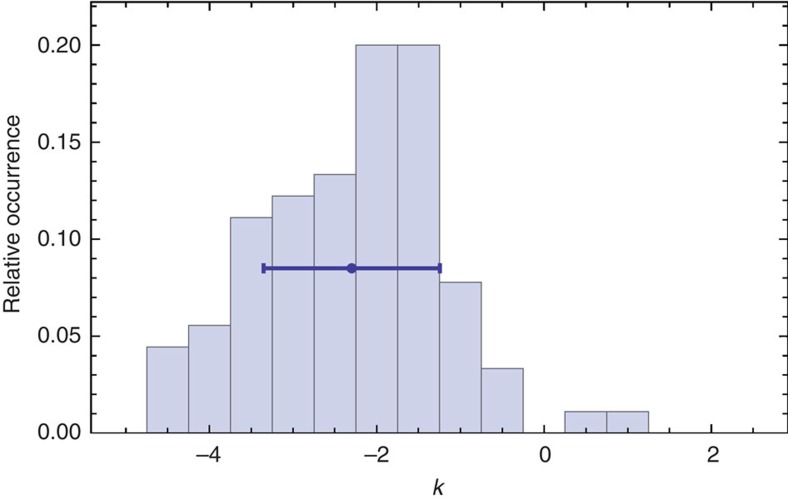
Estimated coefficients. Depicted is the histogram of best estimated coefficients *k* in the local approximation within [*ω*_min_, *ω*_max_] of the spectral density by *I*(*ω*)=*Cω*^*k*^, showing a statistically significant deviation from the Ohmic situation of *k*=1.

The main experimental result is shown in Fig. 3 (see also Supplementary Note 6). The histogram over all optimal power estimates yields *k*=−2.30±1.05, which is a clear deviation from *k*=1 for a locally Ohmic bath density, hence signifying a remarkably strong departure from Markovianity. It is well known that an Ohmic spectral density leads in the weak coupling and high-temperature regimes to Markovian dynamics^13^,^27^. To further strengthen our analysis, we further make this link quantitative. We show that a deviation from a local Ohmic spectral density—which is precisely what is observed—leads to quantifiable non-Markovian dynamics.

### Quantifying non-Markovian harmonic dynamics

Formally, open system dynamics is precisely Markovian if the time evolution is captured by 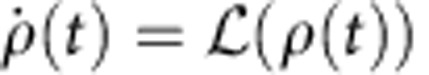, with being a Liouvillian. In order for it to give rise to a valid quantum channel and hence to quantum dynamics, it has to take the so-called Lindblad form,


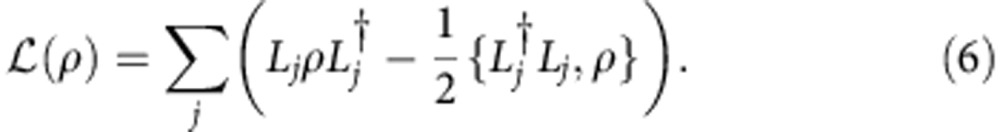


Obviously, any conceivable dynamics that is not generated by Hamiltonian evolution will only be approximately Markovian. This approximation can, however, be exceedingly good. The channels resulting from Markovian dynamics are infinitely divisible^29^,^38^. For harmonic systems, the exact master equation governing time evolution is of the form


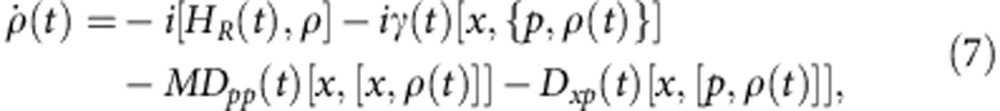


with a time-dependent Hamiltonian *H*_*R*_ and time-dependent coefficients *D*_*pp*_ and *D*_*xp*_ (refs 13, 26, 27). Note the absence of a memory kernel when written in this form, which is implicit in the coefficients.

There are several closely related meaningful ways to quantify Markovianity of a process^29^,^30^,^31^, all essentially deriving from infinite divisibility of the dynamical map (physically originating from short bath correlation times). In precisely this spirit, we capture non-Markovianity by the extent to which the right hand side of equation 7 deviates from a valid Lindblad generator (a rigorous treatment is presented in Supplementary Note 3). The measure taken is


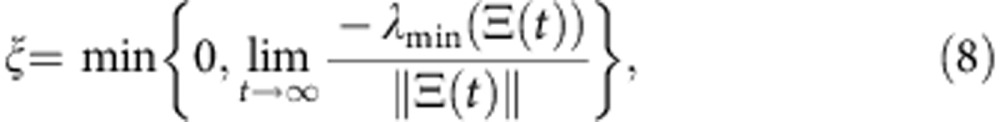



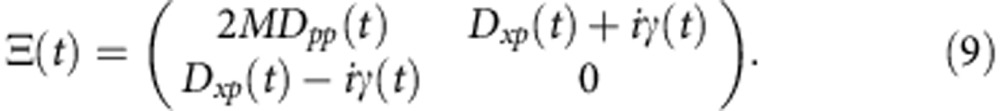


For an Ohmic spectral density with high frequency cutoff, we find that *D*_*xp*_(∞) is very close to zero; in fact, *ξ* is of the order of 10^−15^ for all other parameters chosen as in the experiment. However, our result for the slope at *I*(Ω) gives a lower bound of *ξ*>1.1 × 10^−6^. This shows that the dynamics sharply deviates from a Markovian one. In other words, our analysis unambiguously shows that the heat bath of the micromechanical oscillator is not consistent with Markovian damping of a quantum harmonic oscillator in the high temperature limit.

## Discussion

While we do not expect effects of finite-dimensional bath components resulting, for example, from two-level fluctuators, to measurably influence the result^39^,^40^,^41^, we cannot rigorously exclude such contributions. We can yet strictly and unambiguously falsify the common assumption of a harmonic Ohmic heat bath. Our specific situation is rather described by highly sub-Ohmic damping. We strongly emphasize that our analysis does not rely on any assumption about the resonator geometry. We may, however, still speculate as to why this strongly sub-Ohmic damping is being found. It seems plausible that the specific geometry of the slab used contributes to this non-orthodox decoherence. Indeed, sub-Ohmic spectral densities have been computed in a phononic mode analysis of low-dimensional slabs^18^. However, we also expect intrinsic decoherence mechanisms to be relevant.

It is known that in non-Ohmic baths the coefficients of the master equation governing the dynamics are becoming strongly time-dependent^26^. This means that, while the steady-state properties of a mechanical system may be modified only in a mild way—the deviations of the measured spectrum from Equation 4 for Ohmic spectral densities are small—one should expect larger deviations for predictions in time-dependent situations^42^. It has been pointed out recently that such non-Markovian quantum noise can significantly influence the ability to generate quantum entanglement^43^. Indeed, intricate memory effects come into play in case of non-Markovian dynamics, giving rise to a picture of decoherence beyond basic rate equations.

Finally, our findings complement related research in mechanical engineering. It is known that damping due to internal materials losses can be vastly different from a purely velocity-dependent damping term as typically assumed for a simple harmonic oscillator. Specific models for such non-viscous damping, a prominent model being that of ‘structural' or frequency-independent damping^44^, have been extensively studied in the context of both gravitational wave detection^44^,^45^,^46^ and measurements of the gravitational constant^47^,^48^, where thermal noise in the DC tail of a mechanical resonance poses limits on the achievable sensitivity. In turn, while the accurate measurement of internal friction and the analysis of their origin remains a challenging task, broadband thermal noise measurements have become an important input for the design and engineering of high-*Q* micro- and nanomechanical resonators^49^. This is also important for macroscopic systems such as end mirrors for optical reference cavities or gravitational wave detectors.

Our approach adds two new aspects: first, our analysis provides a direct link to ‘Markovianity'^29^,^30^,^31^ as a statistical property of the environment of a quantum harmonical oscillator. Second, we exploit the enhancement of the thermal noise in the vicinity of the mechanical resonance, instead of probing thermal noise over a broad frequency band. This provides a local estimate of the thermal bath characteristics, which is the relevant property for non-Brownian dynamics. In a next step, combining this method with a sweep in resonance frequency^50^ could provide direct, full broadband mechanical spectroscopy of the thermal bath spectral density, in a ‘tomographic approach'. Our system identification approach is also model-independent, that is, we do not make any prior assumptions on the underlying nature of the dissipation or on the specific shape of the thermal noise spectral density (other than assuming harmonicity). Although the current study is performed at room temperature, in the ‘classical' regime, it can be directly applied to other mechanical resonators that operate close to or in the quantum regime^17^,^51^,^52^,^53^.

In summary, we have introduced a versatile method to directly probe the spectral density of the heat bath of a micromechanical resonator. We demonstrate that the common assumption of Markovian Brownian motion does not hold. This opens the way towards systematic studies of individual dissipation channels such as two-level fluctuators^39^,^40^. In combination with the possibility to geometrically modify the phonon spectrum^18^,^54^,^55^,^56^,^57^, this would allow for full reservoir engineering of quantum harmonic oscillators. We hope that the present work stimulates such further experimental analysis of unorthodox decoherence phenomena opening up alongside technological development.

## Additional information

**How to cite this article**: Gröblacher, S. *et al.* Observation of non-Markovian micromechanical Brownian motion. *Nat. Commun.* 6:7606 doi: 10.1038/ncomms8606 (2015).

## Supplementary Material

Supplementary Information Supplementary Notes 1-6 and Supplementary References
