# Supplementary material for: Observation of non-Markovian micromechanical Brownian motion
Source: Nat Commun. 2015 Jul 28;6:7606. doi: 10.1038/ncomms8606 (PMC4525213; doi:10.1038/ncomms8606)
Supplement: Supplementary Information — Supplementary Notes 1-6 and Supplementary References [file ncomms8606-s1.pdf]

Supplementary note 1: Noise-noise correlations for the damped mode

In this work, we allow for and discuss an arbitrary harmonic model for arbitrary spectral densities of the bath, where special emphasis is put on the weak coupling and high temperature limit, but no further assumptions are being made. We start from the Hamiltonian of the Caldeira-Leggett-model (or Ullersma model) for quantum Brownian motion [1–3], so the standard quantum mechanical damped oscillator,

$$H_m = \frac{p^2}{2m} + \frac{1}{2}m\Omega^2 q^2 + \sum_n \left(\frac{p_n^2}{2m_n} + \frac{1}{2}m_n\omega_n^2 q_n^2\right) + q\sum_n c_n q_n. \tag{1}$$

At this point no assumption is made with respect to the coupling except from it being linear. The equations of motion of the canonical coordinate of the distinguished oscillator are given by

$$\ddot{q}(t) + \Omega^2 q(t) + \frac{2}{m}\int_0^t ds\eta(t-s)q(s) = \frac{f(t)}{m}. \tag{2}$$

In this equation, the inhomogeneity is given by

$$f(t) = -\sum_n c_n \left(q_n(0)\cos(\omega_n t) + \frac{p_n(0)}{m_n}\frac{\sin(\omega_n t)}{\omega_n}\right), \tag{3}$$

whereas the damping kernel η is

$$\eta(s) = \frac{d}{ds}\nu(s), \tag{4}$$

$$\nu(s) = \int_0^\infty d\omega \frac{I(\omega)}{\omega}\cos(\omega s), \tag{5}$$

in terms of the spectral density

$$I(\omega) = \sum_n \delta(\omega-\omega_n)\frac{c_n^2}{2m_n\omega_n}. \tag{6}$$

In the Ohmic regime, so for a spectral density linear in ω until a finite but large cut-off frequency $\Lambda > 0$, this damping kernel is for large Λ arbitrarily well approximated by an expression of the form

$$\eta(t) = \gamma(\infty)m\delta'(t), \tag{7}$$

so Eq. (2) takes a form local in time. Returning to the general case, the exact two-point correlation functions of the thermal force are found to be

$$\langle f(t)f(s)\rangle = \int_{-\infty}^\infty e^{i\omega(t-s)}\frac{\hbar}{2}I(\omega) \times \left(\coth\left(\frac{\hbar\omega}{2k_BT})\right) - 1\right)d\omega, \tag{8}$$

for $t, s \geq 0$, where for simplicity of notation we have extended the definition of the spectral density to $I : \mathbb{R} \to \mathbb{R}^+$ by taking $I(-\omega) = -I(\omega)$.

This expression, valid in general without any approximation, can be cast into a time-local form, albeit the dynamics being non-Markovian. For this, one has to formulate the Green's function $G : \mathbb{R} \to \mathbb{R}$ of the problem. One arrives at a differential equation of the form

$$\ddot{q}(t) + \Omega^2(t)q(t) + \gamma(t)\dot{q}(t) = \frac{\bar{f}(t)}{m} \tag{9}$$

for $t \geq 0$, where now the time-dependent damping is found to be

$$\gamma(t) = \frac{G(t)\dddot{G}(t) - \dot{G}(t)\ddot{G}(t)}{\dot{G}(t)^2 - G(t)\ddot{G}(t)}, \tag{10}$$

and

$$\Omega^2(t) = \frac{\ddot{G}(t)^2 - \dot{G}(t)\dddot{G}(t)}{\dot{G}(t)^2 - G(t)\ddot{G}(t)}. \tag{11}$$

The inhomogeneity becomes

$$\bar{f}(t) = \left(\partial_t^2 + \gamma(t)\partial_t + \Omega(t)^2\right)\int_0^t G(t-s)f(s)ds. \tag{12}$$

It can now be shown, and is physically plausible, that these quantities take their asymptotic values for large times,

$$\lim_{t\to\infty}\gamma(t) = \gamma(\infty), \lim_{t\to\infty}\Omega(t) = \Omega(\infty). \tag{13}$$

These expressions are exact and no approximations have been made until this point. For large times t, the expression

$$\ddot{q}(t) + \Omega^2(\infty)q(t) + \gamma(\infty)\dot{q}(t) \tag{14}$$

is arbitrarily well approximated by $\bar{f}(t)/m$. In fact, the correlation function of this modified driving can readily be found by going into the Fourier domain, with convention taken

$$\tilde{F}(\omega) = \int_{-\infty}^\infty F(t)e^{-i\omega t}dt. \tag{15}$$

An evaluation of this expression reveals that the Fourier transform of the Green's function is given by

$$\tilde{G}(\omega) = \frac{1}{-\omega^2 + 2\hat{\eta}(\omega)/m + \Omega^2}, \tag{16}$$

where we have defined $\hat{\eta}$ and analogously $\hat{\nu}$ as

$$\hat{\eta}(\omega) = \int_0^\infty dt\eta(t)e^{-i\omega t}. \tag{17}$$

The expected two-time correlation function of $\bar{f}$ is then computed to be

$$\langle\bar{f}(t)\bar{f}(s)\rangle = \int_{-\infty}^\infty e^{i\omega(t-s)}\frac{\hbar}{2}I(\omega)\left(\coth\left(\frac{\hbar\omega}{2k_BT}\right) - 1\right) \times ((\Omega(\infty)^2 - \omega^2)^2 + \gamma(\infty)\omega^2)|\tilde{G}(\omega)|^2 d\omega \tag{18}$$

$$= \int_{-\infty}^\infty e^{i\omega(t-s)}\frac{\hbar}{2}I(\omega) \times \frac{(\Omega(\infty)^2 - \omega^2)^2 + \gamma(\infty)\omega^2}{(\Omega^2 - \omega^2 + 2\mathrm{re}(\hat{\eta}(\omega))/m)^2 + (2\mathrm{im}(\hat{\eta}(\omega))/m)^2} \times \left(\coth\left(\frac{\hbar\omega}{2k_BT}\right) - 1\right)d\omega, \tag{19}$$

for $t, s \geq 0$. We also find in terms of ν, rather than in η,

$$\langle\bar{f}(t)\bar{f}(s)\rangle = \int_{-\infty}^\infty \frac{(\Omega(\infty)^2 - \omega^2)^2 + \gamma^2(\infty)\omega^2}{\left(K^2 - \omega^2 + 2\omega\mathrm{im}(\hat{\nu}(\omega))/m\right)^2 + \left(2\omega\mathrm{re}(\hat{\nu}(\omega))/m\right)^2} \times \frac{\hbar}{2}I(\omega)\left(\coth\left(\frac{\hbar\omega\beta}{2}\right) - 1\right)e^{i\omega(t-s)}d\omega, \tag{20}$$

where

$$K^2 = -\frac{2}{m}\nu(0) + \Omega^2, \tag{21}$$

and $\beta = 1/(k_BT)$. This is still an exact expression. The real part is now identified to be

$$\mathrm{re}(\hat{\nu}(\omega)) = \frac{\pi I(\omega)}{2\omega}. \tag{22}$$

The weak coupling approximation amounts to approximating

$$\Omega(\infty)^2 \approx K^2, \gamma(\infty) \approx \frac{\pi I(K)}{mK}, \tag{23}$$

which is true if

$$\left|\frac{2}{m}\partial_\omega\hat{\nu}(\omega)|_{\omega=K}\right| \ll 1, \tag{24}$$

$$\left|\frac{1}{m}\hat{\nu}(K)\right| \ll K \tag{25}$$

and if the imaginary part is negligible. This is the standard weak coupling limit [4], which is the only approximation made in the discussion of quantum Brownian motion. Note that this weak coupling limit does not require the coupling to be so weak for the rotating wave approximation or the 'quantum

optical limit' (see Subsection) to be valid. With these approximations, one finds that

$$\frac{(\Omega(\infty)^2-\omega^2)^2+\gamma^2(\infty)\omega^2}{\left(K^2-\omega^2+\omega\frac{2}{m}\mathrm{im}(\hat{\nu}(\omega))\right)^2+\left(\omega\frac{2}{m}\mathrm{re}(\hat{\nu}(\omega))\right)^2} \tag{26}$$

is extraordinarily well approximated by unity, meaning that the contribution of the spectral density to the two-time correlation function of $\bar{f}$ originates only from the nominator. This is basically the reason, why in Eq. (20) $I(\omega)$ appears only in the numerator. Within this approximation, we have that $\langle\bar{f}(t)^2\rangle \approx \langle f(t)^2\rangle$ to a very good approximation.

Supplementary note 2: Relating the spectra of the mirror and the output light

We now discuss the situation where the harmonic oscillator described above is coupled to a laser field within an optical cavity. The vibrational mode of a high reflective micro-mirror is modelled as a damped harmonic oscillator as in Eq. (1). The micro-mirror together with another solid mirror forms an optical cavity, which is driven by a laser beam. The total Hamiltonian of a harmonic oscillator coupled to thermal bath and laser field within a driven optical cavity is given by

$$H = H_m + \hbar\omega_c a^\dagger a - \hbar g_0 a^\dagger a q + i\hbar E(a^\dagger e^{-i\omega_0 t} - a e^{i\omega_0 t}), \tag{27}$$

where a is the annihilation operator of the optical mode, $g_0 = \omega_c/L$ is the coupling constant of the mechanical to the optical mode. ω_c is the resonance frequency of the cavity with length L and decay rate κ. H_m is the complete Hamiltonian of the distinguished mechanical mode with its environment as in Eq. (1).

$$|E| = \sqrt{2W\kappa/(\hbar\Omega)}, \tag{28}$$

where W is the input power of the laser with frequency ω_0. The Heisenberg picture equations of motion formulated in the interaction picture with respect to $\hbar\omega_0 a^\dagger a$ become, suppressing time dependence,

$$\dot{q} = \frac{p}{m}, \tag{29}$$

$$\dot{p} = -m\Omega^2 q - \sum_n c_n q_n + \hbar g_0 a^\dagger a, \tag{30}$$

$$\dot{a} = -(\kappa + i\Delta_0)a + ig_0 aq + E + \sqrt{2\kappa}a^{\mathrm{in}}, \tag{31}$$

$$\dot{q}_n = \frac{p_n}{m_n}, \tag{32}$$

$$\dot{p}_n = -m_n\omega_n^2 q_n - c_n q, \tag{33}$$

where $\Delta_0 = \omega_c - \omega_0$. In the weak-coupling limit of the previous subsection, the equations of motion turn into

$$\ddot{q} + \gamma(\infty)\dot{q} + \Omega(\infty)^2 q = \frac{\bar{f}}{m} + \frac{\hbar g_0}{m}a^\dagger a, \tag{34}$$

$$\dot{a} = -(\kappa + i\Delta_0)a + ig_0 aq + E + \sqrt{2\kappa}a^{\mathrm{in}} \tag{35}$$

again suppressing time dependence. Based on these expressions, one can proceed exactly as presented in Ref. [5], with the Ohmic bath being replaced by this general thermal bath. In order to progress, it is helpful to make use of dimensionless quantities,

$$Q = \frac{q}{l}, P = \frac{pl}{\hbar}, l = \sqrt{\hbar/(m\Omega(\infty))} \tag{36}$$

and to define $G_0 = g_0 l$. Following Ref. [5], one arrives at an expression which is for large times t, s well approximated by

$$\langle\delta Q(t)\delta Q(s)\rangle \approx \int_{-\infty}^{\infty}\frac{d\omega}{2\pi}|\chi_{\mathrm{eff}}^{\Delta}(\omega)|^2\left(S_{\mathrm{th}}(\omega) + S_{\mathrm{rp}}(\omega,\Delta)\right)e^{i\omega(t-s)}, \tag{37}$$

for the deviations from the asymptotic steady state value

$$\delta Q = Q - \lim_{t\to\infty}\langle Q\rangle = Q - \frac{G_0|\alpha_s|^2}{\Omega(\infty)}, \tag{38}$$

where

$$S_{\mathrm{th}}(\omega) = \frac{\pi I(\omega)}{m\Omega(\infty)}\left(\coth\left(\frac{\hbar\omega}{2k_B T}\right) - 1\right), \tag{39}$$

$$S_{\mathrm{rp}}(\omega,\Delta) = \frac{2\kappa G_0^2|\alpha_s|^2}{\Delta^2+\kappa^2+\omega^2+2\Delta\omega}, \tag{40}$$

$$\chi_{\mathrm{eff}}^{\Delta}(\omega) = \Omega(\infty)\left(\Omega(\infty)^2 + i\gamma(\infty)\omega - \omega^2 - \frac{2G_0^2|\alpha_s|^2\Delta\Omega(\infty)}{\Delta^2+(\kappa+i\omega)^2}\right)^{-1} \tag{41}$$

Here, Δ and α_s are implicitly defined as

$$\Delta = \Delta_0 - \frac{G_0^2|\alpha_s|^2}{\Omega(\infty)}, \alpha_s = \lim_{t\to\infty}\langle a\rangle = \frac{E}{\kappa+i\Delta}. \tag{42}$$

This is different from the expression in Ref. [5] in that both the thermal noise spectrum S_{th} as well as the radiation pressure noise spectrum S_{rp} are being altered.

The final step is to include the actual measurement of the opto-mechanical system and how the information about the motion of the mechanics can be obtained from the measurement of the light leaking out of the cavity. As before, this entire apparatus is assumed to be well-characterised and known, which is a very reasonable assumption for the present experiment.

In the experiment the light leaking out of the optical cavity, referred to as 'signal', is measured by homodyne detection. This means that signal is mixed on a 50:50 beam-splitter with a second, much stronger laser beam. This second beam has the same frequency as the light driving the cavity and is referred to as the 'local oscillator'. The intensities measured in both arms are then electronically subtracted. The result is a voltage, which apart from delta-correlated measurement noise is proportional to some general quadrature of the signal, depending on the phase between the signal and the local oscillator and is, apart from noise, proportional to the corresponding intracavity quadrature. In the the parameter regime relevant here, it turns out that the output quadrature δY^{out} is proportional to δQ subjected to additional noise. Hence, by measuring this quadrature of the signal, information about the mechanical motion and therefore about the thermal bath driving the mechanics can be extracted.

Again following Ref. [5], one arrives in the regime that is experimentally relevant at the expression for the spectrum of the output light measured with quantum efficiency $\zeta > 0$ that is very well approximated by the expression in dimensionless units

$$S_{\delta Y^{\mathrm{out}}}(\omega) \approx \frac{8k_B T\pi\zeta G_0^2|\alpha_s|^2\Omega(\infty)}{m\hbar\kappa}\frac{I(\omega)}{\omega\left((\Omega(\infty)^2-\omega^2)^2+(\gamma(\infty)\omega)^2\right)}. \tag{43}$$

That is to say, by detecting the output light, one can immediately obtain information on the spectral density I of the unknown decohering environment.

Supplementary note 3: Non-Markovian dynamics

For what follows, we will put properties of spectral densities into contact with non-Markovian features of the resulting dynamics. In order to make this link precise, we will first discuss how Markovian and non-Markovian dynamics can be captured for harmonic systems. Generally speaking, there are several closely related meaningful ways to quantify the non-Markovianty of a process [6–8], all essentially deriving from infinite divisibility of the dynamical map, the latter being defined as

$$\rho \mapsto T_t(\rho) = \rho(t) \tag{44}$$

for states ρ. The mathematical property of infinite divisibility can be interpreted in physical terms: Markovian dynamics is forgetful dynamics, one that results from an interaction with a heat bath that does not keep a memory, a property that in turn is originating from short bath correlation times. Fully Markovian dynamics is always an abstraction, even though often, dynamics can be Markovian to an extraordinarily good approximation. By virtue of Lindblad's theorem [9, 10], Markovian dynamics is reflected by a time evolution governed by a master equation

$$\frac{d}{dt}\rho(t) = \mathcal{L}(\rho(t)), \tag{45}$$

with a time-independent generator of so-called Lindblad form,

$$\mathcal{L}(\rho) = \sum_j \left(L_j \rho L_j^\dagger - \frac{1}{2}\{L_j^\dagger L_j, \rho\}\right). \quad (46)$$

Note that in some recent treatments of Markovianity, even time dependent equations of motion are considered Markovian – since we are interested in the long-time limit here, this distinction is of no relevance for our purposes, however. In the focus of attention are harmonic systems of a single mode, coupled to a harmonic bath [11].

Starting point of the analysis is the exact master equation for quantum Brownian motion for an arbitrary spectral density in a general environment [2], given by

$$\begin{aligned}\frac{d}{dt}\rho(t) =& - i\,[H_R(t), \rho(t)] - i\gamma(t)\,[x, \{p, \rho(t)\}]\\ &- MD_{pp}(t)\,[x, [x, \rho(t)]] - D_{xp}(t)\,[x, [p, \rho(t)]]\,.\end{aligned}$$

The time-dependent coefficients are completely determined by the spectral density, however in general in an extraordinarily complicated way. Note that for harmonic systems, the non-Markovian character and the memory are implicitly entirely incorporated by the time dependence of the coefficients of the master equation, and no further memory kernel is necessary to keep generality. As this equation is quadratic in the canonical coordinates, one can easily deduce the dynamical law for the 2×2 real covariance matrix Γ with time dependent entries

$$\Gamma(t) = \begin{pmatrix} 2\langle x^2\rangle(t) & \langle xp + px\rangle(t) \\ \langle xp + px\rangle(t) & 2\langle p^2\rangle(t)\end{pmatrix}, \quad (47)$$

as

$$\frac{d}{dt}\Gamma(t) = -h(t)\Gamma(t) - \Gamma(t)h^T(t) + D(t), \quad (48)$$

$$h(t) = \begin{pmatrix} 0 & -\frac{1}{M} \\ M\Omega_r^2(t) & 2\gamma(t)\end{pmatrix},$$

$$D(t) = \begin{pmatrix} 0 & -D_{xp}(t) \\ -D_{xp}(t) & 2MD_{pp}(t)\end{pmatrix}. \quad (49)$$

One can characterise the entire interaction with the environment by a Hermitian 2×2 matrix Γ that one can deduce from this differential equation. For this, we represent $H_R(t)$ as the quadratic form

$$H_R(t) = \begin{pmatrix} x & p \end{pmatrix} h_R(t) \begin{pmatrix} x \\ p \end{pmatrix} \quad (50)$$

and define the symplectic matrix as

$$\sigma = \begin{pmatrix} 0 & 1 \\ -1 & 0 \end{pmatrix}. \quad (51)$$

It then follows that

$$h^T(t) = (2h_R(t) - \mathrm{im}(\Xi(t)))\,\sigma, \quad (52)$$

$$D(t) = \sigma^T \mathrm{re}(\Xi(t))\sigma. \quad (53)$$

We find that the system-environment interaction is then specified by

$$\Xi(t) = \begin{pmatrix} 2MD_{pp}(t) & D_{xp}(t) + i\gamma(t) \\ D_{xp}(t) - i\gamma(t) & 0\end{pmatrix}. \quad (54)$$

The long-time dynamics is Markovian if and only if $\Xi(\infty)$ corresponds to a master equation in Lindblad form, which in this case is equivalent with $\Xi(\infty)$ being positive semi-definite. Hence, a meaningful measure of non-Markovianity is – in case of long-time non-Markovian dynamics – the absolute value of the smallest eigenvalue of $\Xi(\infty)$, normalised by the operator norm of $\Xi(\infty)$. More precisely, the measure used is

$$\xi = \min\left\{0, \lim_{t\to\infty} \frac{-\lambda_{\min}(\Xi(t))}{\|\Xi(t)\|}\right\}. \quad (55)$$

This quantity, which is particularly transparent in this case of harmonic dynamics, can easily be related to other measures of non-Markovianity discussed in the literature [6–8, 11]. If $\xi = 0$, the dynamics is Markovian in the long-time limit. Otherwise, it is non-Markovian, and more significantly so the larger this quantity. As $\lim_{t\to\infty} D_{pp}(t) > 0$, one finds the simple expression

$$\xi = \frac{(1+\mu)^{1/2} - 1}{(1+\mu)^{1/2} + 1}, \quad (56)$$

with

$$\mu = \lim_{t\to\infty} \frac{D_{xp}^2(t) + \gamma^2(t)}{M^2 D_{pp}^2(t)}. \quad (57)$$

Since ξ is a simple monotonous function of μ, one can as well quantify non-Markovianity of the long-time dynamics in terms of μ.

Supplementary note 4: Relating non-Markovianity to spectral densities

In this subsection, we relate notions of non-Markovianity of dynamics with properties of spectral densities. This link has already been rather well established [2, 12], in that it is well known that the high-temperature Ohmic setting corresponds to the Markovian limit to a very good approximation. Here, in order to further strengthen the claim of the main text, we significantly extend this link and make it more quantitative and precise: We show how a deviation from this behaviour can be directly and quantitatively related to non-Markovian features. That is to say, we make the link between Ohmic spectral densities and Markovian dynamics in the above precise sense quantitative.

Ref. [2] presents the following simplified expressions for the coefficients in the weak-coupling limit

$$\delta\Omega^2\,(t) = 2\int_0^t ds\eta(s)\cos{(\Omega s)}\,,\;\; \Omega^2(t) = \Omega^2 + \delta\Omega^2(t), \quad (58)$$

$$\gamma\,(t) = -\frac{1}{\Omega}\int_0^t ds\eta(s)\sin{(\Omega s)}\,, \quad (59)$$

$$D_{xp}\,(t) = \frac{1}{\Omega}\int_0^t dsn(s)\sin{(\Omega s)}\,, \quad (60)$$

$$mD_{pp}\,(t) = \frac{1}{\Omega}\int_0^t dsn(s)\cos{(\Omega s)}\,. \quad (61)$$

The noise and damping kernel (in a notation adapted to the present work) are given by

$$n(s) = \int_0^\infty d\omega I(\omega)\coth\left(\frac{\hbar\omega}{2k_BT}\right)\cos{(\omega s)}\,, \quad (62)$$

$$\eta(s) = \frac{d}{ds}\left(\int_0^\infty d\omega\frac{I(\omega)}{\omega}\cos{(\omega s)}\right) = -\int_0^\infty d\omega I(\omega)\sin{(\omega s)}\,. \quad (63)$$

So far, we have merely recapitulated properties of general quantum Brownian motion in the weak coupling limit. We now turn to making the link of a deviation from the high-temperature Ohmic setting to non-Markovian dynamics precise. In order to do so in a most transparent fashion, we first state the mild and natural assumptions made on the spectral density $I : \mathbb{R}^+ \to \mathbb{R}^+$. We make the following assumptions:

(i) The function

$$\omega \mapsto I(\omega)\coth\left(\frac{\hbar\omega}{2k_BT}\right) \quad (64)$$

is in $L^1\,([0,\infty))$.

(ii) The spectral density can be approximated in a vicinity of Ω by an affine function (or a power law), i.e., it is not rapidly oscillating.

The first assumption is necessary in order to get a well-defined noise kernel, such that the occurring integrals are convergent. The second assumption is required for the statistical analysis. In addition to these assumptions on the spectral density, we will invoke the above weak coupling and a high temperature approximation. Both the weak coupling as well as the high temperature approximation are valid to overwhelming accuracy for the experiment at hand. A standard calculation shows that

$$\lim_{t\to\infty}\gamma(t) = \gamma(\infty) = \frac{\pi}{2\Omega}I(\Omega), \quad (65)$$

$$\lim_{t\to\infty} mD_{pp}(t) = mD_{pp}(\infty) = \frac{\pi}{2\Omega}I\,(\Omega)\coth\left(\frac{\hbar\Omega}{2k_BT}\right). \quad (66)$$

The computation of $\lim_{t\to\infty} D_{xp}(t)$ is, however, more subtle and we will make use of assumption (ii). We find

$$D_{xp}(t) = \frac{1}{\Omega}\int_0^t ds \left(\int_0^\infty d\omega I(\omega)\coth\left(\frac{\hbar\omega}{2k_BT}\right)\cos(\omega s)\right)\sin(\Omega s)\,. \tag{67}$$

By assumption (i), we may use Fubini's theorem to get

$$\begin{aligned} D_{xp}(t) &= \frac{1}{\Omega}\int_0^\infty d\omega I(\omega)\coth\left(\frac{\hbar\omega}{2k_BT}\right) \\ &\times \frac{1}{2}\left\{-\frac{\cos((\Omega+\omega)s)}{\Omega+\omega}\Big|_0^t - \frac{\cos((\Omega-\omega)s)}{\Omega-\omega}\Big|_0^t\right\} \\ &= \frac{1}{2\Omega}\int_0^\infty d\omega I(\omega)\coth\left(\frac{\hbar\omega}{2k_BT}\right)\left(\frac{1}{\Omega+\omega}\right. \\ &+ \left.\frac{1}{\Omega-\omega}(1-\cos((\Omega-\omega)t)) - \frac{1}{\Omega+\omega}\cos((\Omega+\omega)t)\right). \end{aligned} \tag{68}$$

If

$$\omega \mapsto I(\omega)\coth\left(\frac{\hbar\omega}{2k_BT}\right) \tag{69}$$

is in $L^1([0,\infty))$, by assumption (i), then so is

$$I(\omega)\coth\left(\frac{\hbar\omega}{2k_BT}\right)\frac{1}{\omega+\Omega}. \tag{70}$$

Therefore, for $t \to \infty$ the last term vanishes by the Riemann-Lebesgue Lemma. We cannot proceed similarly for the other oscillatory part because $1/x$ is not integrable over $\mathbb{R}^+$. However, as $\lim_{x\to\infty}(1-\cos(x))/x = 0$ the integral is convergent and we can investigate the influence of the local behaviour of the spectral density around the resonance frequency. We proceed by splitting the domain of integration into a part

$$U_\Omega = (\Omega-\delta, \Omega+\delta) \tag{71}$$

close to resonance and its complement for a small $\delta > 0$. We can then again make use of the Riemann-Lebesgue Lemma for the oscillatory part over $\mathbb{R}^+ \setminus (\Omega-\delta,\Omega+\delta) =: U_\Omega^c$, to obtain

$$D_{xp}^{\mathrm{res}}(t) = \frac{1}{2\Omega}\int_{\Omega-\delta}^{\Omega+\delta} d\omega I(\omega)\coth\left(\frac{\hbar\omega}{2k_BT}\right) \tag{72}$$

$$\times\left\{\frac{1}{\Omega+\omega} + \frac{1}{\Omega-\omega}(1-\cos((\Omega-\omega)t))\right\}, \tag{73}$$

$$D_{xp}^{\mathrm{off}}(\infty) = \int_{U_\Omega^c} d\omega I(\omega)\coth\left(\frac{\hbar\omega}{2k_BT}\right)\frac{1}{\Omega^2-\omega^2}. \tag{74}$$

Choosing $\delta > 0$ sufficiently small, and invoking assumption (ii), we can approximate the spectral density around Ω by an affine function

$$\omega \mapsto I(\Omega) + Ck\Omega^{k-1}(\omega-\Omega), \tag{75}$$

as the local affine approximation of $\omega \mapsto C\omega^k$, for $k \in \mathbb{R}$. In this approximation, and approximating in the high temperature limit in which $\coth(\hbar\omega/(2k_BT))$ is approximated by $2k_BT/(\hbar\omega)$, the limit $t \to \infty$ can be performed. In this approximation, we get

$$\begin{aligned} &\frac{1}{2\Omega}\int_{\Omega-\delta}^{\Omega+\delta} d\omega I(\omega)\coth\left(\frac{\hbar\omega}{2k_BT}\right)\left(\frac{1}{\Omega-\omega}(1-\cos((\Omega-\omega)t))\right) \\ &\overset{t\to\infty}{\longrightarrow} \frac{I(\Omega)}{\beta\hbar\Omega^2}(1-k)\ln\left(\frac{\Omega+\delta}{\Omega-\delta}\right). \end{aligned} \tag{76}$$

The other contribution of D_{xp}^{res} depends negligibly on the local power law and only on the value of the spectral density at Ω itself,

$$\begin{aligned} &\frac{1}{2\Omega}\int_{\Omega-\delta}^{\Omega+\delta} d\omega \frac{I(\omega)\coth(\hbar\omega/(2k_BT))}{\Omega+\omega} = \frac{I(\Omega)}{\beta\hbar\Omega^2}\times \\ &\left(\ln\left(\frac{(\Omega-\delta/2)(\Omega+\delta)}{(\Omega+\delta/2)(\Omega-\delta)}\right) + \underbrace{k\ln\left(\frac{(\Omega+\delta/2)^2}{(\Omega-\delta/2)^2}\frac{(\Omega-\delta)}{(\Omega+\delta)}\right)}_{\text{negligible}}\right). \end{aligned} \tag{77}$$

The second term is several orders of magnitude smaller than the first for parameters similar to the ones of the present experiment, and is hence negligible. Up to this point, the assumptions (i)-(ii) as well as the weak coupling and high temperature limits have been invoked.

So far we have shown that the dependence on the affine function locally approximating a power law $\omega \mapsto C\omega^k$, is essentially of the form $D_{xp} = a + b(1-k)$, with

$$a = D_{xp}^{\mathrm{off}}(\infty) + \frac{I(\Omega)}{\beta\hbar\Omega^2}\ln\left(\frac{(\Omega-\delta/2)(\Omega+\delta)}{(\Omega+\delta/2)(\Omega-\delta)}\right), \tag{78}$$

$$b = \frac{I(\Omega)}{\beta\hbar\Omega^2}\ln\left(\frac{\Omega+\delta}{\Omega-\delta}\right). \tag{79}$$

As it turns out, the only negative contribution to a comes from the term

$$\int_{\Omega+\delta}^\infty d\omega I(\omega)\coth\left(\frac{\hbar\omega}{2k_BT}\right)\frac{1}{\Omega^2-\omega^2}. \tag{80}$$

For values $k < 0$ and not too oscillatory spectral density I, one can safely assume that $a > 0$. This is a very mild, but strictly speaking an additional assumption about the spectral density being not too oscillatory outside the relevant window U_Ω.

In conclusion this shows that then

$$D_{xp} \geq \frac{I(\Omega)}{\beta\hbar\Omega^2}(1-k)\ln\left(\frac{\Omega+\delta}{\Omega-\delta}\right). \tag{81}$$

We are finally in the position to have an expression at hand from which we can readily read off a lower bound to ξ, the measure of non-Markovianity at stationarity for long times $t \to \infty$, depending only on measurable quantities. One finds that the μ that defined the measure of non-Markovianity ξ as in Eq. (56) is bounded from below by

$$\mu \geq \frac{4}{\pi^2}(1-k)^2\ln\left(\frac{\Omega+\delta}{\Omega-\delta}\right)^2; \tag{82}$$

it is straightforward to see that a bound to μ also gives rise to a bound to ξ. This is significantly larger than zero and orders of magnitudes larger than the value for $k = 1$, corresponding to the Ohmic case. In this sense, we can make a precise and quantitative link between a local deviation from the Ohmic case $k = 1$ and the resulting non-Markovian dynamics. Often, it is read in the literature that the Ohmic case corresponds to Markovian dynamics: Here, this connection is made quantitative.

To further strengthen this point, we make a model for a spectral density of

$$I(\omega) = \begin{cases} \frac{I(\Omega)}{\Omega}\omega, & \omega \in [0,\Omega-\delta), \\ \frac{I(\Omega)}{\Omega^k}\omega^k, & \omega \in [\Omega-\delta,\Omega+\delta), \\ \frac{I(\Omega)}{\Omega}\omega, & \omega \in [\Omega+\delta,\Lambda), \\ 0, & \omega \in [\Lambda,\infty). \end{cases} \tag{83}$$

for $k \in \mathbb{R}$, $\delta > 0$, and cut-off frequency $\Lambda = 10^7\Omega$. For this generic spectral density

$$D_{xp} = \frac{I(\Omega)}{\beta\hbar\Omega^2}(1-k)\ln\left(\frac{\Omega+\delta}{\Omega-\delta}\right), \tag{84}$$

up to a term merely logarithmically divergent in Λ which is negligibly small for reasonable cut-off frequencies . Then, $k = 1$ precisely corresponds to a negligible measure of non-Markovianity, that is, to Markovian dynamics. Having said that, the link between spectral densities that can locally be approximated by power laws or affine functions and non-Markovian dynamics is more generally valid, as pointed out above.

Supplementary note 5: Impact of technical noise

Several potential sources of technical noise in our experimental setup exist that could in principle influence the frequency dependence of the observed noise-floor and might have an impact on our experimental results. The emission from the laser itself, as well as the electro-optical modulator and the photo-detectors could introduce excess, frequency dependent noise, which however can easily be verified experimentally by measuring the light spectrum of the reflected laser field from an unlocked, far off-resonant optomechanical cavity. In the frequency band of interest, the noise floor is more

than one order of magnitude below the thermal noise floor of the mechanical resonator and shows negligible frequency dependence, which is reflected in our experimental error bars. We further rule out noise from our optomechanical cavity, by measuring the same cavity without a mechanical element (see Fig. 2). This is done by moving to a mirror pad on the chip that is not released. In addition, electronic noise in our detection system plays a negligible role.

Finally, higher order mechanical modes could influence the frequency dependence of our measured noise floor. We have simulated our device and find that these modes do not influence the noise floor in the frequency window we are interested in any significant way. Note that any impact from higher order modes would make the coupling to the environment more supra-Ohmic rather than sub-Ohmic, as observed in our experiment.

Supplementary note 6: Statistical analysis

In this section we describe the statistical analysis in more detail. We process raw data in the form of samples from a time series $\{\delta Y^{\rm out}(t)\}$ obtained from the homodyning measurement with a 10 MHz sampling rate. From this, $m = 9,000$ batches of data are being formed, each containing $n = 100.000$ data points. For every such batch, the data are Fourier transformed, to get data in the frequency domain. In this frequency domain, the mean of 100 data sets is considered. In this way, 90 independently distributed spectra are obtained. For each of these 90 Fourier transforms, the optimal power k in the power law

$$\omega \mapsto C\omega^k \tag{85}$$

is being determined by fixing an interval $[\omega_{\rm min}, \omega_{\rm max}]$ around the renormalised resonance frequency $\Omega(\infty)$, to minimise the mean square deviation. For the present experiment,

$$\omega_{\rm min} = 885 \text{ kHz}, \tag{86}$$

$$\omega_{\rm max} = 945 \text{ kHz} \tag{87}$$

have been chosen. As this minimisation of the mean square deviation constitutes a non-convex optimisation problem, a global simulated annealing algorithm is used. Fig. 3 depicts the outcome of this analysis. Again, while for each realisation of a spectrum several values of k are approximately compatible with the data, one can estimate the optimal k with high significance from the complete data set.

To further corroborate these findings, many variants of the above statistical estimation have been systematically explored. Notably, several instances of bootstrapping, involving a resampling of data, give rise to findings that are indistinguishable from the above ones. The results are largely independent against a different choice of the frequency window or the way batches are being chosen. Also, the findings are not different when not the least squares difference is being considered in the actual intensities, but the logarithms thereof.

Supplementary references

[1] Caldeira, A. O. & Leggett, A. J. Path integral approach to quantum brownian motion. *Physica A* **121**, 587–616 (1983).

[2] Hu, B. L., Paz, J. P. & Zhang, Y. Quantum Brownian motion in a general environment: Exact master equation with nonlocal dissipation and colored noise. *Phys. Rev. D* **45**, 2843–2861 (1992).

[3] Martinez, E. A. & Paz, J. P. Dynamics and thermodynamics of linear quantum open systems. *Phys. Rev. Lett.* **110**, 130406 (2013).

[4] Fleming, C. H. & Hu, B. L. Non-Markovian dynamics of open quantum systems: Stochastic equations and their perturbative solutions. *Ann. Phys.* **327**, 1238 (2012).

[5] Genes, C., Vitali, D., Tombesi, P., Gigan, S. & Aspelmeyer, M. Ground-state cooling of a micromechanical oscillator: generalized framework for cold damping and cavity-assisted cooling schemes. *Phys. Rev. A* **77**, 033804 (2008).

[6] Wolf, M. M., Eisert, J., Cubitt, T. S. & Cirac, J. I. Assessing non-Markovian dynamics. *Phys. Rev. Lett.* **101**, 150402 (2008).

[7] Laine, E.-M., Piilo, J. & Breuer, H.-P. Measure for the degree of non-Markovian behavior of quantum processes in open systems. *Phys. Rev. Lett.* **103**, 210401 (2009).

[8] Rivas, A., Huelga, S. F. & Plenio, M. B. Entanglement and non-Markovianity of quantum evolutions. *Phys. Rev. Lett.* **105**, 050403 (2010).

[9] Lindblad, G. *Commun. Math. Phys.* **48**, 119 (1976).

[10] Gorini, V., Kossakowski, A. & Sudarshan, E. *J. Math. Phys.* **17**, 821 (1976).

[11] Heinosaari, T., Holevo, A. S. & Wolf, M. M. The semigroup structure of Gaussian channels. *Quantum Inf. Comp.* **10**, 0619 (2010).

[12] O'Connell, A. D. *et al.* Quantum ground state and single-phonon control of a mechanical resonator. *Nature* **464**, 697–703 (2010).
